# Colorectal cancer: the facts in the case of the microbiota

**DOI:** 10.1172/JCI155101

**Published:** 2022-02-15

**Authors:** Slater L. Clay, Diogo Fonseca-Pereira, Wendy S. Garrett

**Affiliations:** 1 Department of Immunology and Infectious Diseases and Department of Molecular Metabolism, Harvard T.H. Chan School of Public Health, Boston, Massachusetts, USA.; 2 Harvard T.H. Chan Microbiome in Public Health Center, Boston, Massachusetts, USA.; 3 Broad Institute of MIT and Harvard, Cambridge, Massachusetts, USA.; 4 Department of Medical Oncology, Dana-Farber Cancer Institute, Boston, Massachusetts, USA.; 5 Harvard Medical School, Boston, Massachusetts, USA.

## Abstract

The importance of the microbiota in the development of colorectal cancer (CRC) is increasingly evident, but identifying specific microbial features that influence CRC initiation and progression remains a central task for investigators. Studies determining the microbial mechanisms that directly contribute to CRC development or progression are revealing bacterial factors such as toxins that contribute to colorectal carcinogenesis. However, even when investigators have identified bacteria that express toxins, questions remain about the host determinants of a toxin’s cancer-potentiating effects. For other cancer-correlating bacteria that lack toxins, the challenge is to define cancer-relevant virulence factors. Herein, we evaluate three CRC-correlating bacteria, colibactin-producing *Escherichia coli*, enterotoxigenic *Bacteroides fragilis*, and *Fusobacterium nucleatum*, for their virulence features relevant to CRC. We also consider the beneficial bioactivity of gut microbes by highlighting a microbial metabolite that may enhance CRC antitumor immunity. In doing so, we aim to elucidate unique and shared mechanisms underlying the microbiota’s contributions to CRC and to accelerate investigation from target validation to CRC therapeutic discovery.

The microbiome’s relevance to the initiation and progression of colorectal cancer (CRC) is increasingly appreciated, but defining how microorganisms influence susceptibility to and progression of cancer remains a challenge. Data from cross-sectional epidemiological studies, unbiased microbiome profiling of stool and colorectal tissues, and preclinical models have uncovered specific taxa and bacterial factors such as toxins that contribute to colorectal cancer. Identification of microbial features associated with CRC is important, but host and bacterial factors must be carefully considered to gain mechanistic insights into a microbe’s contributions to carcinogenesis. Bacterial virulence is a concept that often includes infection of a host cell and damage to host cells and tissues. However, some microorganisms discussed here may not invade host cells but rather activate host cell receptors or coat the surface of a tumor in a patchy film. Furthermore, with the exceptions of a few notable organisms, e.g., *Helicobacter pylori*, it is difficult to directly causally implicate microorganisms in carcinogenesis in the absence of permissive host features, e.g., oncogenic mutations. As such, assessing factors of CRC-associated microbes that influence tumor development requires careful consideration and a broad definition of virulence.

While specific “oncomicrobes” (microorganisms directly associated with malignancy) warrant research and therapeutic targeting, a “one taxon, one target” approach risks oversimplifying the complexity of colorectal carcinogenesis and limiting understanding of the composite features of a given microbiome. Acknowledging this limitation, herein we evaluate three CRC-correlating bacteria and their virulence features. We begin with an examination of polyketide synthase–expressing (*pks*+) *Escherichia coli*, enterotoxigenic *Bacteroides fragilis* (ETBF), and *Fusobacterium nucleatum* (*Fn*), before highlighting a microbial metabolite of potential benefit for CRC antitumor immunity. We focus on these oncomicrobes because compelling data from preclinical studies and patient-based investigations support their roles in CRC susceptibility or progression. Many bacteria, including oral microbes, have been identified in colonic and stool-based studies of CRC; but ETBF, *Fn*, and *pks*^+^ *E. coli* have emerged as warranting deeper consideration for colorectal carcinogenesis (1–3). Given that bacteria often have co-conspirators or work in concert as part of a community, these three exemplars are not the only microbes important for CRC, but they provide insight into targetable mechanisms of action in CRC.

## *Pks*^+^* E*. *coli*

*E. coli* are a highly prevalent, but not very abundant, Gram-negative facultative anaerobe of the distal gastrointestinal tract. *E*. *coli* are a vast and diverse group of bacteria including more than 700 identified serotypes. While many *E*. *coli* are harmless to humans, Shiga toxin–producing, enterohemorrhagic *E*. *coli* cause significant morbidity and mortality (4). Other pathogenic *E*. *coli* that cause diarrheal diseases include enterotoxigenic, enteropathogenic, enteroaggregative, and adherent and invasive *E*. *coli*, the latter of which has been implicated in the pathogenesis of ileal Crohn’s disease *E*. *coli* harbor genotoxins including cytotoxic necrotizing factor, cytolethal distending toxin, cycle-inhibiting factor, and colibactin, a DNA-damaging secondary metabolite produced by its *pks* island. Additionally, Enterobacteriaceae family members, including *Citrobacter koseri*, *Klebsiella pneumoniae*, and *Enterobacter aerogenes*, can produce colibactin (5, 6).

**Figure 1:**
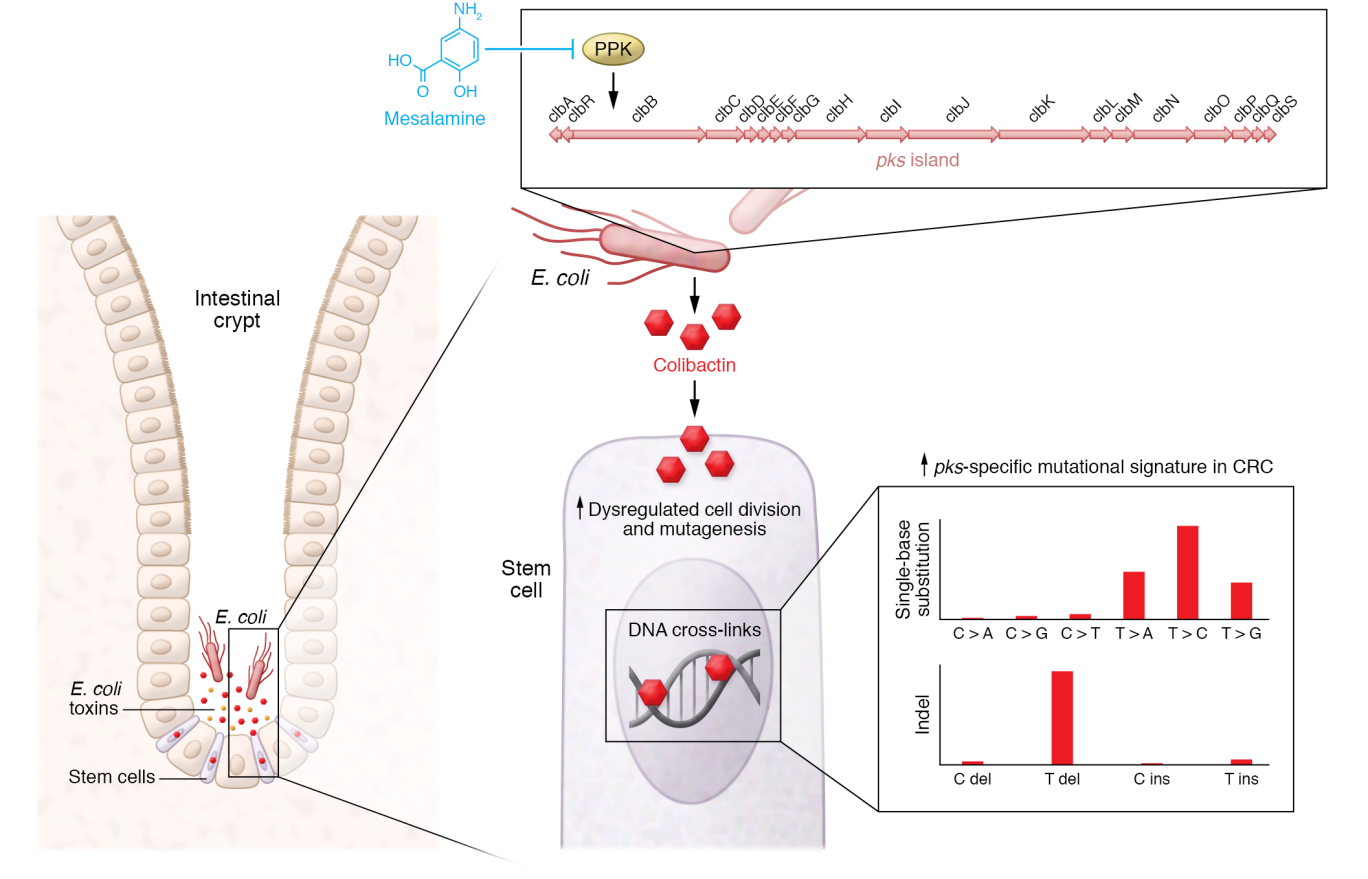
Potential mutagenic effects of *pks^+^ E*. *coli*. *E*. *coli* strains can produce harmful toxins. Top right: The polyketide synthase (*pks*) island encodes the genes required for the synthesis of colibactin, a well-known genotoxin. Recent studies showed that polyphosphate kinase (PPK) activity is essential for ClbB function and colibactin metabolism. The ulcerative colitis medication mesalamine reduces PPK activity and colibactin production. Bottom right: Colibactin binding to DNA forms DNA cross-links and interstrand breaks that dysregulate cell division and increase mutagenesis. Importantly, a colibactin-specific mutational signature, characterized by single-base substitutions, deletions, and insertions at T sites, is enriched in CRC. indel, insertion-deletion; del, deletion of nucleotide; ins, insertion of nucleotide.

Colibactin derived from *pks*^+^ *E*. *coli* induces DNA double-strand breaks and interstrand DNA cross-links (Figure 1 and refs. 7–10). Colibactin-producing *E*. *coli* are positively associated with CRC, with an approximately 60% detection rate in CRC patients and approximately 20% in healthy individuals (11–16). These *E*. *coli* also increase tumor burden in murine models of CRC (13, 14, 17, 18). However, a thorough understanding of colibactin-CRC links has been slowed by knowledge gaps in colibactin’s structure and biosynthesis. Components of the colibactin biosynthesis pathway are encoded by 19 *clb* genes within the *pks* pathogenicity island, and the numerous precursors and instability of bioactive colibactin have hindered efforts to isolate and characterize the genotoxin (7, 8, 19, 20).

Building on previous studies showing that colibactin forms DNA cross-links (9, 10), two groups developed techniques using DNA as probes to isolate and identify bioactive colibactin, characterize its structure, and resolve its biosynthesis. Using isotope labeling, DNA adductomics, and mass spectrometry, researchers demonstrated that colibactin comprises dual cyclopropane warheads, which form DNA cross-links by alkylating adenine residues (Figure 1 and refs. 21, 22). Together, these biochemical approaches provide mechanistic insight into *pks*^+^ *E. coli*–mediated mutagenesis.

While *pks*^+^ *E. coli* appear to influence CRC by colibactin-mediated mutagenesis, the specific mutations that result from colibactin exposure have only recently been revealed. In a recent study, Pleguezuelos-Manzano et al. leveraged human intestinal organoids to demonstrate that *pks*^+^ *E*. *coli* induce a CRC-associated mutational signature (23). Researchers administered *pks*^+^ *E*. *coli* to organoids via periodic luminal injections and determined the mutational accumulation in epithelial cells exposed to this bacterium, independent of microbiota- or immune-mediated effects. Whole-genome sequencing (WGS) of organoid subclones revealed that cells exposed to functional *pks*^+^ *E*. *coli* but not *clbQ*-deficient *E*. *coli*, which cannot synthesize colibactin, accumulated a distinct pattern of somatic mutations. The mutational pattern includes thymine (T) insertions at T homopolymers and increased T>N single-base substitutions (SBSs), preferentially occurring in adenine-rich regions. These SBSs are consistent with mutations triggered by colibactin’s dual cyclopropane warheads forming cross-strand links at adenine residues (10, 22, 24).

After identifying a *pks*-specific mutational pattern in their organoid platform, researchers analyzed WGS data from more than 5000 human cancers to determine whether the *pks* signature matched mutations in human tumors. Data from two patient cohorts showed that the *pks*-specific mutational signature was present in human tumors and was particularly enriched in CRC. Importantly, this mutational pattern was identified in 112 known CRC driver mutations. *APC* (adenomatous polyposis coli), the most mutated gene in CRC, harbored the greatest number of mutations matching the *pks* signature (>5%). Other studies have identified colibactin-specific somatic mutations in a survey of several thousand CRC genomes (25), and in genes involved with p53 signaling (26). Furthermore, somatic mutations consistent with the *pks* signature have previously been identified in healthy human colon biopsies and were linked to mutagenesis that occurred during early childhood (27). This suggests that colibactin may be an important driver of mutations that increase the risk of CRC later in life, representing an early life event and potential modifiable risk factor.

Since *pks*^+^ *E. coli* are found in approximately 20% of apparently healthy individuals (12, 14), unanswered questions remain as to why these bacteria drive carcinogenesis in some individuals but not others. Unanswered questions also remain about *E*. *coli* strain Nissle 1917, which is marketed as a “probiotic” yet harbors a *pks* island, regarding whether it induces a similar mutational signature. Other important questions involve how *pks* genes are regulated, what environmental cues promote colibactin production, what is the importance of temporal and biogeographical factors (e.g., exposure to *pks*^+^ *E*. *coli* in the colonic crypt near the stem cell compartment, as depicted in Figure 1), what level of *pks*^+^ *E*. *coli* bacterial load is problematic, and whether there are critical interactions with other microbial virulence factors within the tumoral or luminal microbiota that influence colibactin’s effects on CRC development.

Systematic mutational studies support that 14 of the 19 genes encoded by the *pks* island are required for *pks*-mediated genotoxicity (7). While the colibactin biosynthesis pathway has been characterized (10, 18, 19, 28), and expression of *pks* genes is upregulated in murine models and CRC patients (14, 15), the factors that regulate expression or suppression of *pks* genes remain understudied. Given the complexities of the *pks* molecular assembly line, questions remain about whether factors produced in addition to colibactin could mitigate or exacerbate the effects ascribed to it. Experiments such as profiling of conditioned media from *clbQ*-deficient and -sufficient *E*. *coli* in conjunction with cell-based DNA adduct or mutation screening assays may identify bioactive analytes that modulate colibactin’s mutagenic effects.

Despite these knowledge gaps, targeting of *pks*-encoded products within the colibactin synthesis pathway, such as ClbM and ClbP, reduces genotoxicity in vitro and tumor burden in vivo (29, 30). Intriguingly, a recent study determined that polyphosphate kinase (PPK) is essential for ClbB’s role in colibactin metabolism, and targeting PPK with mesalamine, a medication frequently prescribed for ulcerative colitis, reduces colibactin production (Figure 1 and ref. 31). Collectively, basic, clinical, and bioinformatic approaches have revealed that colibactin directly drives CRC-associated mutations. Questions of how, where, and when to intervene regarding *pks*^+^ *E*. *coli* to make an impact in CRC prevention loom large, but colibactin inhibitors seem a near-future treatment avenue.

## Enterotoxigenic *Bacteroides fragilis*

*B. fragilis* is an early and prevalent colonizer of the human colon; vertical transmission has been reported, and over 30% of infants harbor intestinal *B*. *fragilis* at 3 months of age (32–36). This Gram-negative anaerobic bacterium has substantial strain diversity in the human gut based on isolate sequencing as well as metagenomic analyses (37). While *B*. *fragilis* phylogeny can be described in multiple ways, strains can be categorized as toxigenic or non-toxigenic. Non-toxigenic strains have been extensively investigated in terms of their immunomodulatory roles (38, 39); and non-toxigenic *B*. *fragilis* may be enriched in the earliest stages of colorectal tumorigenesis and influence neoplastic progression (40).

**Figure 2:**
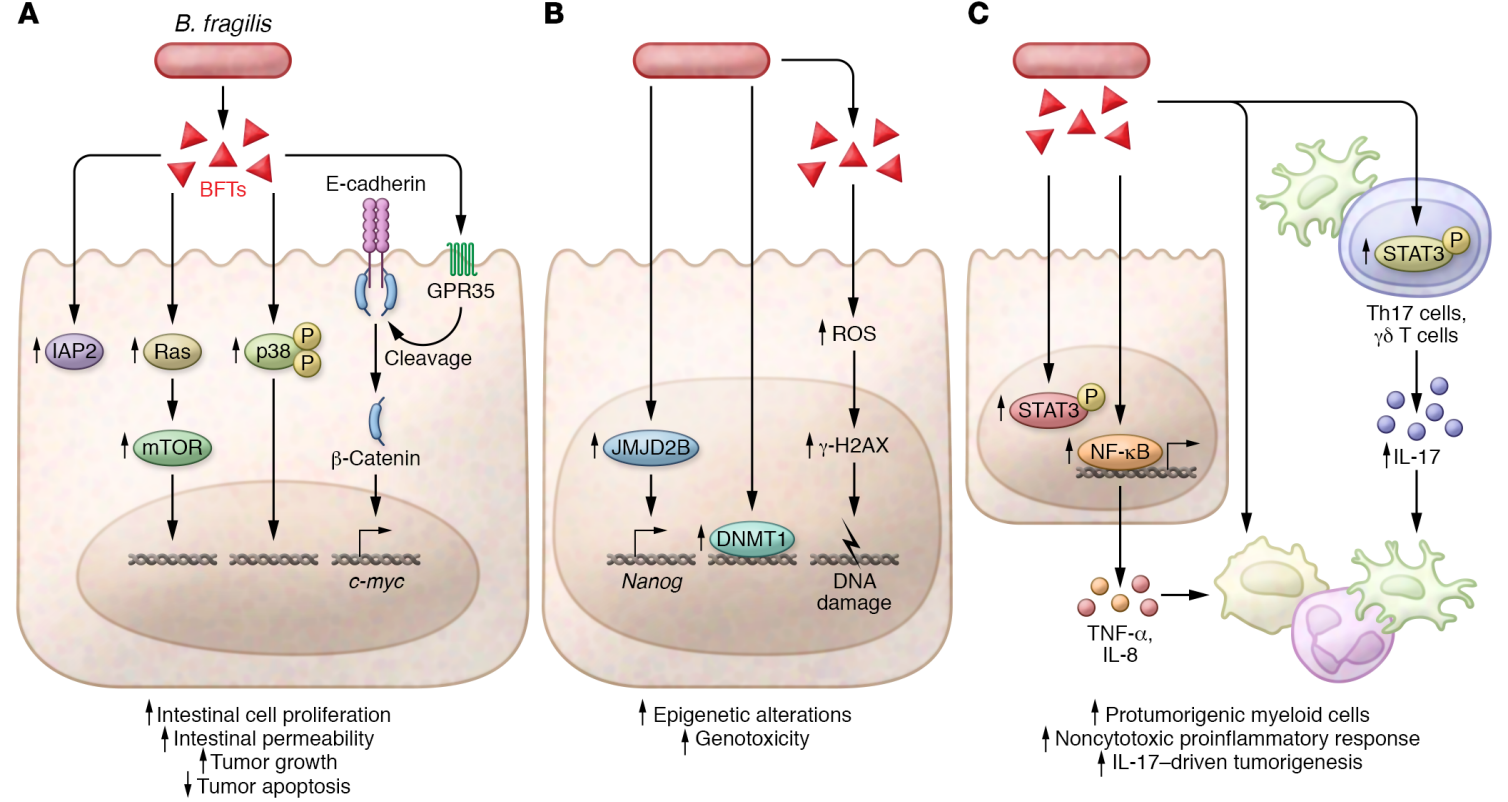
Enterotoxigenic *Bacteroides fragilis* promotes tumorigenesis by distinct mechanisms. (**A**) *B*. *fragilis* toxins (BFTs) activate the Ras/mTOR and p38 mitogen-activated protein kinase (p38) intracellular signaling pathways. BFTs induce inhibitor of apoptosis protein-2 (IAP2) expression, resulting in increased tumor growth and inhibition of apoptosis. BFTs also increase intestinal cell proliferation and permeability by inducing c-myc expression after E-cadherin cleavage and β-catenin nuclear localization, in a process that was recently shown to involve G protein–coupled receptor 35 (GPR35). (**B**) Enterotoxigenic *B*. *fragilis* (ETBF) promotes epigenetic alterations with the potential to cause DNA damage by inducing DNA methyltransferase 1 (DNMT1) recruitment and inducing JmjC domain–containing histone demethylase 2B (JMJD2B) in CRC cells. ETBF-produced BFTs also induce DNA damage by increasing ROS generation. (**C**) ETBF and BFTs induce a proinflammatory environment that contributes to carcinogenesis. BFTs induce activation of the transcription factors STAT3 and NF-κB, increasing intestinal permeability and production of inflammatory cytokines. In a multistep process, ETBF induces phosphorylation (“P” in yellow circles) of STAT3 and IL-17–producing Th17 and γδ T cells. Both processes promote the recruitment of pro-tumorigenic myeloid cells that suppress cytotoxic antitumor immunity.

In contrast to non-toxigenic strains, enterotoxigenic *B*. *fragilis* (ETBF) are associated with inflammatory bowel diseases and CRC (13, 41–45). ETBF induce colitis and tumorigenesis in murine models of CRC, including *Apc^MinΔ716/+^* mice, which are a genetic model of familial adenomatous polyposis (FAP), and the azoxymethane/dextran sodium sulfate (AOM/DSS) model of colitis-associated cancer (13, 46–48). Pathogenicity in these models is dependent on *B*. *fragilis* toxin (BFT), a 20 kDa matrix metalloprotease that includes three isoforms: BFT-1, BFT-2, and BFT-3 (49, 50). *Bft-1* and *Bft-2* are detectable in CRC clinical samples and are abundant in the mucosa during late-stage disease (42, 43, 51). As a pleiotropic virulence factor, BFT acts on colonic epithelial cells (CECs) to initiate multiple downstream pathways that can promote tumorigenesis (Figure 2). BFT stimulates CEC proliferation, suppresses apoptosis, induces epigenetic alterations, and drives immune dysregulation. Collectively, these effects can promote a pro-carcinogenic setting for the initiation and progression of CRC.

One of the earliest observed effects of BFT on host cells was the cleavage of membrane-bound E-cadherin from CECs (52). E-cadherin cleavage triggers β-catenin nuclear localization and signaling, which induces c-myc expression and sustained epithelial cell proliferation (53, 54). Another mechanism of increased proliferation is through the induction of *B*. *fragilis*–associated long noncoding RNA 1 (BFAL1), which activates the Ras homolog in the mammalian target of rapamycin (mTOR) pathway, and increases CRC tumor growth (55). BFT also inhibits epithelial cell apoptosis by inducing expression of cellular inhibitor of apoptosis protein-2 (c-IAP2) (56, 57). Recently, BFT was shown to suppress apoptosis by upregulating sulfiredoxin-1 (Srx-1) and MAPK expression (58). Thus, there are multiple pathways of ETBF-mediated hyperproliferation and apoptotic suppression (Figure 2A), but how BFT elicits pro-tumorigenic effects in CECs has been elusive. BFT signals through the CEC-expressed GPR35 (59). Pharmaceutical antagonists, shRNA-mediated interference, and CRISPR/Cas9–mediated knockout demonstrate that targeting of GPR35 reduced E-cadherin cleavage and pathology of ETBF-induced murine colitis. Thus, GPR35 signaling may be an important pathway that contributes to ETBF pathogenesis.

While hyperproliferation is a hallmark of tumorigenesis, epigenetic modifications are also essential contributors to cancer development (60). ETBF induces a range of epigenetic modifications in CECs that may initiate DNA damage (Figure 2B). In *Apc^MinΔ716/+^* mice colonized with ETBF, both gene silencing in CpG islands and DNA methyltransferase 1 (DNMT1) recruitment increase (61, 62). BFT also enhances chromatin accessibility of AP-1/ATF transcription factor binding sites (63). Furthermore, in coculture experiments, ETBF upregulates JmjC domain–containing histone demethylase 2B (JMJD2B) in CRC cell lines, which promotes expression of Nanog homeobox (NANOG), an important transcription factor for stemness (63, 64). Besides epigenetic modifications, BFT elicits generation of reactive oxygen species, inducing DNA damage and activating histone γ-H2AX, which is indicative of DNA repair (56). Taken together, these studies provide evidence that ETBF drives a wide range of epigenetic modifications and genotoxicity.

In addition to ETBF-mediated pro-tumorigenic effects in CECs, the contribution of a proinflammatory immune response is a critical factor for carcinogenesis (Figure 2C). Engagement of BFT with CECs leads to increased permeability of the epithelial barrier, NF-κB signaling, and production of cytokines including IL-8 and TNF-α (52, 53, 65). Then a multistage immune response involving T cells and myeloid cells ensues that is required for the distal colon tumorigenesis observed in the ETBF-*Apc^MinΔ716/+^* model. Early studies of immune response in this model showed that ETBF induces STAT3 and increases Th17 cells and γδ T cells, and blockade of IL-17 or IL-23 inhibited tumorigenesis (50). While targeting IL-17 inhibits tumor formation, tumorigenesis was only abrogated when both Th17 cells and γδ T cells were targeted (66). The specific requirement for IL-17 to drive carcinogenesis, distinct from other inflammatory contexts, was highlighted by a Treg depletion study, which shifted the cytokine profile from an IL-17 to an IFN-γ bias, increasing colitis and decreasing tumor growth (67). T cells are not the only immune cells important for tumorigenesis in this model, as a combination of BFT and IL-17 promotes recruitment and differentiation of polymorphonuclear and mononuclear myeloid cells that suppress T cell differentiation and cytotoxicity (68, 69). Together, these studies highlight a dynamic and site-specific immune response to BFT, whereby Th17 and γδ17 cells produce IL-17, which promotes NF-κB activation and signaling in distal CECs that in turn produce cytokines that drive recruitment and differentiation of pro-tumorigenic myeloid cells.

While the ETBF-*Apc^MinΔ716/+^* model triggers IL-17–dependent tumorigenesis in the distal colon, ETBF induces distinct clinical manifestations and immune responses depending on host genetics. ETBF-colonized *BRAF^V600E^Lgr5^Cre^Min* mice developed tumors localized to the mid-colon, and these tumors shared characteristics with human BRAFV600E tumors, including increased infiltration by CD8^+^ T cells and improved responsiveness to anti–PD-L1 therapy (70). This highlights the critical role of interactions between microbial virulence factors and host genetics in shaping tumorigenesis and responsiveness to immunotherapy. Elucidating host genetics and other factors that regulate the diverse effects of BFT represents a critical and achievable next step, especially given the accessibility of current CRISPR/Cas9 screening tools for both human colorectal cell lines and human colon organoids.

Clinical studies and in vivo models provide insights into the potential for ETBF to drive epigenetic modifications and DNA damage, dysregulate epithelial function, and induce inflammation to promote colorectal carcinogenesis. While BFT promotes carcinogenesis through multiple direct and indirect pathways, ETBF also creates a niche for colonization by other oncobacteria. Dejea et al. characterized bacterial biofilms in FAP patients, predominantly comprising *E*. *coli* and *B*. *fragilis*, and the colonic mucosae of these individuals were enriched for genes encoding BFT (*bft*) and colibactin (*clb*) (13). FAP patient biopsies contained significantly more ETBF and *pks*^+^ *E*. *coli* compared with healthy controls, and higher rates of co-colonization. Turning to in vivo models, researchers determined that monocolonization with either *pks*^+^ *E*. *coli* or ETBF induced few tumors, but co-colonization led to high tumor burden and invasive adenocarcinoma. Notably, tumorigenesis was dependent on expression of BFT and colibactin, as deletions of *bft* or *pks* abrogated tumor development.

The genotoxicity of *pks*^+^ *E*. *coli* requires viable bacteria contacting intestinal epithelial cells (7, 71), so researchers set out to determine whether ETBF exposure increased *pks*^+^ *E*. *coli* colonization of the colonic mucosa. Using an azoxymethane (AOM) model of tumorigenesis, ETBF co-colonization with *pks*^+^ *E*. *coli* reduced colonic mucus thickness, increased *E*. *coli* colonization of the mucosa, increased colibactin-mediated DNA damage, and increased tumor burden. Conversely, co-colonization of ETBF with *E*. *coli^Δpks^* did not increase DNA damage or tumorigenesis, despite increased mucosal colonization (13). These results suggest that ETBF may enhance the genotoxic effects of *pks*^+^ *E*. *coli* by degrading the mucus layer and enabling colibactin to directly contact CECs. Therefore, in addition to direct genotoxicity, disruption of the epithelial barrier, and dysregulation of immune responses, ETBF has the potential to drive CRC by establishing a niche for other oncomicrobes, including *pks*^+^ *E*. *coli*, to promote a pro-tumorigenic environment.

## *Fusobacterium* *nucleatum*

While *pks*^+^ *E*. *coli* and ETBF express toxins, *F*. *nucleatum* (*Fn*) does not, prompting more broad thinking about how this opportunistic bacterium contributes to CRC. *Fn* is a Gram-negative anaerobic bacterium whose natural niche is the human oral cavity. *Fn* are remarkably diverse and consist of four subspecies (*nucleatum*, *animalis*, *vincentii*, and *polymorphum*); each subspecies comprises significant strain diversity (72). *Fn* has been studied extensively in periodontal disease and infections of the oropharynx and placenta. Its enrichment in colorectal tumor tissues versus adjacent normal tissues has ignited a firestorm of articles (73, 74). Investigations of *Fn*’s roles in colon and rectal cancer biology include its use in stool-based CRC screening tests, effects on the tumor microenvironment, potential roles in differential response to treatments, and contribution to overall survival (72). Recent mechanistic studies have delved into defining the host response to *Fn* in preclinical models and elucidating virulence features relevant to CRC.

In considering the Gram-negative bacterial virulence arsenal, secretion systems (SS) constitute important arms and ammunition. To secrete proteins, Gram-negative bacteria use many SS, grouped from I to IX. These SS include both transport/export machinery and biological effectors (75). Effectors can function in environmental sensing, facilitate binding to host tissues, and have diverse virulence roles. Of the nine types of SS, *Fn* subspecies harbor only type V, and not all subspecies do. Type V SS are often referred to as autotransporters and are divided into types Va–Ve (76). Autotransporters are secreted or outer membrane proteins with structural features that facilitate their transport across bacterial membranes and to the bacterial cell surface. Umaña et al. closed and curated nine fusobacteria genomes, including two *Fn* genomes with a focus on type Va SS autotransporters, whose predicted functions include adhesins, proteins that help bacteria adhere to biotic or abiotic surfaces, and serine proteases (77).

**Figure 3:**
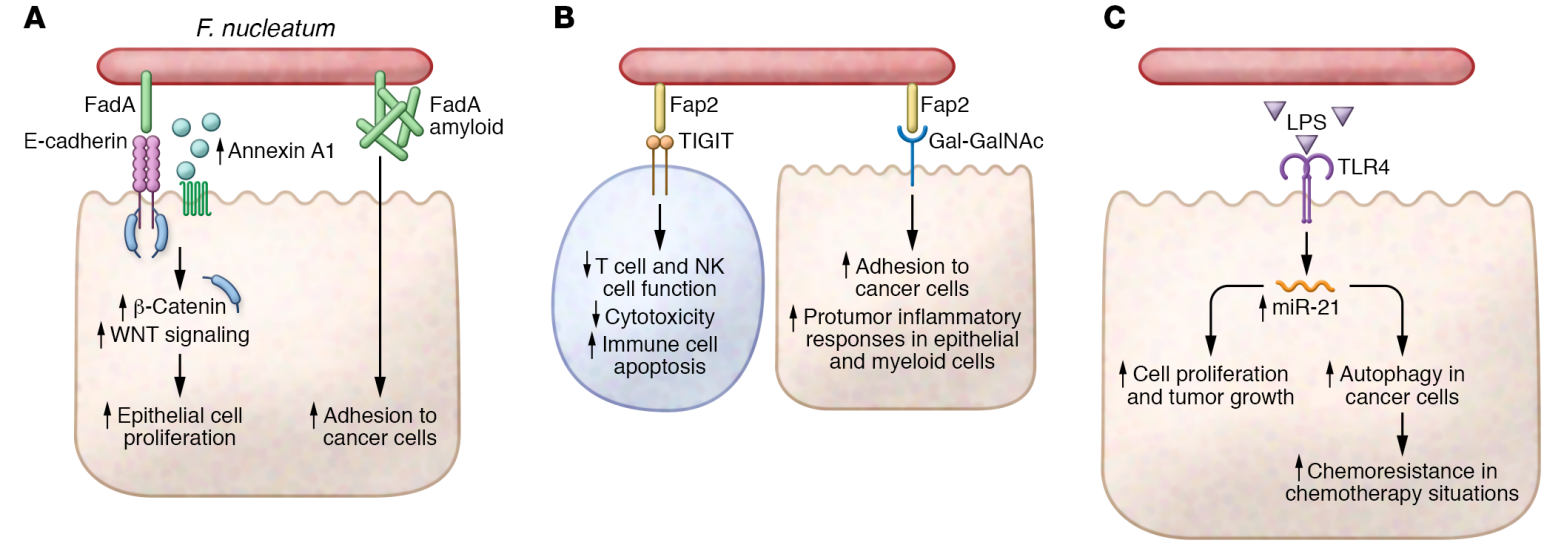
Potential mechanisms of *Fusobacterium* *nucleatum* activity in CRC. (**A**) Fusobacterium adhesin A (FadA) binding to E-cadherin increases β-catenin and WNT signaling and upregulates annexin A1 that drives epithelial cell proliferation. FadA also has amyloid-like properties that enhance *F*. *nucleatum* (*Fn*) adhesion to cancer cells. (**B**) *Fusobacterium* autotransporter protein 2 (Fap2) binds d-galactose-β(1-3)-*N*-acetyl-d-galactosamine (Gal-GalNAc) on cancer cells and recruits *Fn* to tumors. Fap2 also binds to T cell immunoreceptor with Ig and immunoreceptor tyrosine-based inhibitory motif domains (TIGIT) and impairs T and NK cell function, reduces cytotoxicity, and promotes immune cell death, resulting in tumor escape from immunosurveillance. Fap2^+^ *Fn* activates epithelial and myeloid cells and induces a pro-tumorigenic inflammatory response. (**C**) *Fn* LPS induces the expression of microRNA-21 in colon epithelial cells in a TLR4-dependent manner, which results in dysregulated cell proliferation and tumor growth. This same pathway also increases cancer cell autophagy, which enhances resistance to chemotherapy-induced cell death.

Known *Fn* type Va SS adhesins include Fap2, Aim1, RadD, and CmpA. Fap2 is a large galactose-inhibitable adhesin implicated in both colorectal carcinogenesis and preterm birth (78). The d-galactose-β(1-3)-*N*-acetyl-d-galactosamine (Gal-GalNAc) binding domain of Fap2 plays important roles in *Fn* aggregative behavior, facilitating its binding to bacteria and host tissue expressing Gal-GalNAc, especially dysplastic colonic adenomas and colorectal cancers (Figure 3 and refs. 79, 80). Fap2’s Gal-GalNAc lectin activity facilitates its binding to human primary CRC tumors and metastatic deposits (80). When injected intravenously into mice harboring orthotopic colon tumors, Fap2 markedly enhanced *Fn* presence and abundance in the tumors. Fap2 also binds and activates signaling of the immune checkpoint inhibitor TIGIT, blunting antitumor immunity and explicating how *Fn* can impair the function of adaptive CD4^+^ T cells and CD8^+^ T cells (81) and innate lymphocytes, such as NK cells (Figure 3 and ref. 82). While not all *Fn* strains express this large protein (3165 amino acids), many fusobacteria harbor predicted adhesins in their genomes with a high degree of homology to *Fap2* (77).

In contrast with Fap2, RadD and CmpA are considerably smaller adhesins that have predominantly been investigated for their roles in *Fn*’s polymicrobial binding behavior (83, 84). Potentially relevant to colorectal carcinogenesis as relates to colonic tumoral biofilms, *Fn* employs RadD to adhere to *Clostridioides difficile* and enhances *C*. *difficile*’s extracellular polysaccharide production and biofilm formation (85). While less is known about the type Va SS adhesin Aim1, it was studied because of its homology to Fap2, and similar ability to induce apoptosis in Jurkat cells (86). This and another early study showing *Fn*’s immunosuppressive and proapoptotic effects on lymphocytes and myeloid cells (87) foreshadow later work on *Fn*’s myeloid cell immunomodulatory roles in the *Apc^Min/+^* model (88), Fap2’s proinflammatory effects on myeloid cells cocultured with colon cancer cells (89), Fap2’s interactions with TIGIT (81), and *Fn’s* Th17-enhancing functions in the colon (90). Beyond these four adhesins, ten additional type Va SS autotransporters are encoded by *Fn* ATCC 23726 (91, 92). Given that ATCC 23726 is one of the rare genetically tractable *Fn* strains (93, 94), there is the opportunity to better understand the roles of these undercharacterized *Fn* autotransporters in CRC virulence.

*Fn* genomes harbor type Vb, Vc, and Vd autotransporters (77). While little is known about the functional roles of type Vb autotransporters in *Fn* virulence, recent studies shed light on the role of type Vc trimeric autotransporters (77, 95) and type Vd (94). FvcA, a type Vc trimeric autotransporter adhesin, also known as CbpF, binds both carcinoembryonic antigen (CEA), a tumor protein associated with many cancers, including CRC, and CEACAM1 (95), a type 1 membrane receptor protein that is highly expressed in the tumor microenvironment, where it functions in tolerance and exhaustion (96). CbpF1 may inhibit CD4^+^ T cell responses and antitumor immunity by interacting with CEACAM1 (95). While the *Fn* type V autotransporters studied to date are primarily adhesins, they can also function as proteases or lipases, such as the recently characterized FplA (94). This 85 kDa protein binds several phosphoinositide-signaling lipids, and with a knockout strain now available, it will be fascinating to elucidate its role in CRC. Beyond the type V autotransporter adhesins, other adhesins have been studied for their roles in *Fn*’s CRC virulence.

FadA and its homologs FadA2 and FadA3 are a family of small adhesins that are present in the genomes of *Fn* (76). FadA has been studied extensively in *Fn* subsp. *polymorphum* 12230 by Yiping Han’s laboratory, and they have uncovered multiple potential roles of this adhesin in both preterm birth and colorectal carcinogenesis (97). FadA binds E-cadherin expressed on colon cancer cell lines and activates β-catenin signaling critical for oncogenic pathways related to cell growth, proliferation, and polarity (Figure 3 and ref. 97). These investigations follow earlier work demonstrating that FadA could bind E-cadherin on vascular endothelial cells (98). Subsequent studies revealed that FadA’s E-cadherin binding upregulates annexin A1, a WNT/β-catenin signaling modulator important for cyclin D1 activation (99). This observation may explicate the pro-proliferative effects observed with cocultures of strain 12230 and CRC cell lines that express annexin A1. A recent study, also from the Han laboratory with strain 12230, revealed a new perspective on FadA. Extracellular FadA binds Congo red, a sign of amyloid-like properties, and this amyloid-like FadA enhanced *Fn* binding to CRC cell lines in vitro and CRC xenograft growth in vivo (Figure 3 and ref. 100). Similarly to Fap2, FadA appears to be a multifunctional adhesin.

The lipopolysaccharide (LPS) of many bacteria can function as a virulence factor, and *Fn*’s LPS has been reported on extensively. While the majority of *Fn* LPS studies have focused on its role in periodontal pathology, a few papers have focused on host TLR4 and CRC. In a TLR4-dependent manner, *Fn* upregulates microRNA-21 in colon cancer cell lines and CRC tumor models, which enhanced colon cancer cell proliferation and tumor growth (Figure 3 and ref. 100). Engagement of this same pathway activated autophagy in cancer cells, conferring chemoresistance (101). The positive correlation between CRC *Fn* bacterial load and shortened time to progression may relate to activation of these pathways. TLR4 signaling can intersect with many host cell biological pathways, and a recent study found that TLR4 activation led to AKT signaling, downregulating Keap1 and increasing NRF2 to promote transcription of CYP2J2 (102). Identification of this pathway helped explain an observation from a paired metagenomics and lipidomics study showing that high levels of fusobacteria in CRC patient tumors and the presence of *Fn* in DSS-AOM tumors in mice led to increased serum levels of the polyunsaturated fatty acid 12,13-epoxyoctadecenoic acid. In experiments with 12,13-epoxyoctadecenoic acid, the investigators found that it enhanced cancer cell invasion, migration, and tumor size (102). With all these potential mechanisms at play in preclinical models, it seems important to determine which *Fn* strains and behaviors are important to target for prevention and treatment of CRC.

Sequencing studies of CRC led to the discovery of fusobacterial enrichment in CRC, and more recent studies are leading investigators to what *Fn* strains are more prevalent in human CRC (103). Now RNA sequencing studies of *Fn* invading host cells are providing insights into *Fn*’s strategies for invasion and its vulnerabilities. Cochrane et al. provide a highly informative transcriptomic profiling of *Fn* invasion of a well-differentiated colon cancer cell line (104). In identifying *Fn* invasion–specific genes, the authors provide potential biomarkers to study, as well as targets for combating tumoral *Fn*. For example, upregulation of a membrane protein with a reticulocyte binding domain and hemolysin may underlie a mouth-to-bloodstream route for *Fn* colonization of CRC tumoral and metastatic deposits (80, 105). This raises potential opportunities for enhanced preventative dental care with oral *Fn* decontamination in patients with, or at high risk for, CRC. Additionally, invasion leads to changes in *Fn* metabolism. Alterations in *Fn* amino acid metabolism can increase *Fn* short-chain fatty acid production that contributes to an IL-17–mediated proinflammatory, tumor-permissive milieu (90). Thus, considering *Fn* transcriptomes during invasion or within dysplastic and neoplastic sites may provide more targets for CRC prevention and treatment.

With chemoprevention in mind for primary and secondary CRC, Brennan et al. undertook transcriptional profiling studies of *Fn* to elucidate how aspirin was exerting both bacteriostatic and bactericidal effects on *Fn* strains (106). They identified a core set of aspirin-responsive genes, distinct from a general stress response, that reveal underappreciated vulnerabilities and potential antivirulence approaches for *Fn* treatment. Given the importance of making cancer-related data publicly available to facilitate translation, Vogel and colleagues recently generated a series of RNA maps for the *Fn* subspecies and *Fusobacterium* *periodonticum* across various growth conditions, focusing on coding and noncoding RNAs, and developed a data portal to share these data (107). Hopefully, the large amount of data made publicly available in these three studies will facilitate more in-depth study of *F*. *nucleatum* and its contributions to CRC.

## Therapeutic potential for the microbiota in CRC

While specific bacterial taxa and microbe-derived bioactives can promote CRC tumorigenesis, recent research highlights the beneficial potential for the microbiota in cancer. This benefit can occur through restraining tumor growth or enhancing immunotherapy by promoting an immunologically “warm” immune checkpoint inhibitor–responsive (ICI-responsive) tumor phenotype (108, 109). An association between *Bacteroides* species and enhanced anti-CTLA4 efficacy has been reported (110), and *Bifidobacterium* species have been shown to promote anti–PD-1 responsiveness in mouse models of melanoma and in patients (111, 112). Furthermore, the potential to increase ICI efficacy through fecal microbiota transfer (FMT) has been reported by several recent studies (113–115). These findings contributed to the development of two recent FMT clinical trials with metastatic melanoma patients, in which microbiota from ICI-responsive donors were transferred to ICI-refractory recipients. These phase I trials show promise, with clinical responses achieved in 3 of 10 or 6 of 15 patients (116, 117).

While basic and clinical studies highlight an important and targetable role for the microbiota in cancer immunotherapy, identifying the bacteria-derived factors that drive these effects or their mechanisms of action has been a major challenge for the microbiome field. Unfortunately, only a subset of patients who have colon cancer stand to benefit from the ICI therapies. Individuals with colon cancer who have impaired mismatch repair capabilities (microsatellite instability–high [MSI^hi^]) often benefit from ICI, but those with the more common microsatellite-stable (MSS) CRC do not (118–120). The challenge, from a CRC perspective, is to identify how the microbiota or its metabolites can be leveraged to enhance antitumor immunity.

## Inosine’s potential to benefit CRC antitumor immunity

Mager et al. identified inosine as a bacteria-derived metabolite that enhances ICI efficacy in a mouse model of CRC and revealed the mechanism underlying these effects (121). In vivo screening of gnotobiotic mice with bacteria isolated from ICI-treated mouse tumors revealed that *Bifidobacterium pseudolongum* increased intratumoral IFN-γ^+^ CD4^+^ T cells and CD8^+^ T cells, and IFN-γ^+^ CD4^+^ T cells in the spleen. Using serum metabolomics, the investigators found that inosine was selectively and significantly increased in *B*. *pseudolongum* monocolonized mice. Notably, inosine monophosphate and hypoxanthine (an inosine precursor and degradation product, respectively) were identified as elevated in mice colonized with an 11-strain microbial consortium that improves antitumor immune responses (115).

These findings prompted investigators to determine whether inosine enhances antitumor immunity and ICI efficacy in vivo, and if so, how. Inosine is a potent ligand for the adenosine A_2A_ receptor (A_2A_R) (122, 123), and A_2A_R signaling can both inhibit and enhance T cell responses and antitumor immunity (124–126). Inosine’s immune-modulating effects are context dependent, with potential to drive opposing outcomes. Inosine enhanced tumor growth and reduced IFN-γ expression by T cells in anti-CTLA4–treated mice; however, with exogenous CpG added as costimulation, inosine increased T cell expression of IFN-γ, and tumor growth was suppressed. While inosine effects relied on T cell–intrinsic A_2A_R signaling, they also required DCs and IL-12 signaling.

In addition to the heterotopic MC38 model, Mager et al. tested the effect of their ICI-promoting strains in two genetic models of CRC, one MSI^hi^ and the other MSS (121). The ICI-enhancing strains were effective in the MSI^hi^ model but not the MSS model, known to be resistant to anti-CTLA4 therapy. Collectively, this work identifies inosine as a beneficial microbial bioactive that promotes ICI efficacy by signaling through T cell–intrinsic A_2A_R. Besides identifying a potential adjuvant for ICI therapy, this work reveals a nuanced microbiota-dependent immune-modulating pathway that can enhance or inhibit antitumor immunity.

In addition to functioning as a signaling molecule, recent work shows that inosine can enhance antitumor therapy by providing an energy source for T cells in a glucose-restricted environment such as the TME (127). As such, inosine may have multiple modes of action for enhancing antitumor immune responses. These findings offer hope for developing novel therapeutics, but the context-dependent effects of A_2A_R signaling and the multiple effects of microbial metabolites warrant caution about targeting the microbiota when mechanistic understanding is lacking. While research examining associations between the microbiome and cancer treatment will continue to provide insights, future mechanistic studies hold promise to fine-tune and enhance the safety and efficacy of microbiota-derived therapeutics.

## Conclusion

The three microbes we have discussed here share several mechanisms underlying their contributions to a tumor-permissive colonic environment. Colibactin and BFT directly and indirectly cause DNA damage, alter gene expression, and increase proliferation, all of which are central to carcinogenesis. Another shared feature of these oncomicrobes is the promotion of a pro-tumorigenic microbial niche, including biofilms. Fusobacterial adhesins including Fap2, RadD, and FadA use multiple mechanisms to facilitate *Fn* aggregation, adhesion to dysplastic tissue, and biofilm formation. While *Fn* adhesins contribute to host interaction and invasion, their multifunctionality underscores how even well-known virulence features can contribute to CRC in unexpected ways. Beyond adhesins, the multiple functions of virulence factors like BFT, or indeed the beneficial bioactive inosine, highlight that such multifunctionality is ubiquitous, warranting careful consideration when evaluating other microbial factors.

By revealing mechanistic insights into how, instead of just which, microbes promote CRC, research is highlighting molecular targets for preventative and treatment strategies. As causal roles for microbial virulence are validated in CRC, such features become targets for small-molecule therapeutics, biologics, or vaccines. Fap2 is a clear target, given its roles in *Fn* adhesion to CRC tissues, in addition to compromising antitumor immunity. Targeting enzymes in colibactin synthesis, e.g., with ClbP inhibitors, is another approach under evaluation. Increased appreciation of microbial molecular targets has also invigorated research into repurposing existing drugs, like mesalamine and aspirin, to inhibit virulence pathways.

Immune dysregulation is another common feature of oncomicrobe virulence. ETBF and *Fn* both induce a pro-tumorigenic inflammatory milieu, with elevated IL-17, IL-23, and neutrophil numbers. Targeting the IL-17/IL-23 axis with antibodies has had mixed success. While anti–IL-17 therapy has shown promise in psoriatic arthritis patients, these biologics can paradoxically increase intestinal inflammation in ulcerative colitis patients, limiting interest in their utility for CRC patients. Anti–IL-23 therapies have shown promise for treating ulcerative colitis, but the benefit for CRC prevention or therapy is unclear. Furthermore, the effect of targeting IL-23 on the growth of the microbes driving immune dysregulation must be considered.

Taken together, strategies including mining of clinical data, omics, and clinically relevant preclinical models will help move the field from identifying CRC-correlating microbes to identifying factors that contribute to a pro-tumorigenic environment. Transcriptome profiling is now being used to identify such factors; and the identification of an *Fn* membrane protein with a reticulocyte binding domain that may underlie *Fn* colonization of CRC tissue is an encouraging example. In addition to revealing therapeutic targets, microbial virulence features could be incorporated into sequencing assays routinely carried out for tumor mutational testing. This approach has potential to offer diagnostic and prognostic information that can guide treatment of CRC patients and patients with a variety of solid tumors. While the case may be clear that the microbiota has contributory roles in CRC, the next hurdle for the field is to demonstrate that the microbiota and its metabolites can be utilized to offer clinical benefits for CRC patients.

## Version 1 02/15/2022

Electronic publication
